# Role of Cannabinoids in Oral Cancer

**DOI:** 10.3390/ijms25020969

**Published:** 2024-01-12

**Authors:** Brigitte Cretu, Alexandra Zamfir, Sandica Bucurica, Andreea Elena Scheau, Ilinca Savulescu Fiedler, Constantin Caruntu, Ana Caruntu, Cristian Scheau

**Affiliations:** 1 Department of Oral and Maxillofacial Surgery, “Carol Davila” Central Military Emergency Hospital, 010825 Bucharest, Romania; brighy15@gmail.com (B.C.); ma.zamfir23@gmail.com (A.Z.); 2 Department of Gastroenterology, “Carol Davila” University of Medicine and Pharmacy, 020021 Bucharest, Romania; sandica.bucurica@umfcd.ro; 3 Department of Gastroenterology, “Carol Davila” University Central Emergency Military Hospital, 010825 Bucharest, Romania; 4 Department of Radiology and Medical Imaging, Fundeni Clinical Institute, 022328 Bucharest, Romania; andreea.ghergus@gmail.com; 5 Department of Internal Medicine, “Carol Davila” University of Medicine and Pharmacy, 050474 Bucharest, Romania; ilinca.savulescu@umfcd.ro; 6 Department of Internal Medicine and Cardiology, Coltea Clinical Hospital, 030167 Bucharest, Romania; 7 Department of Physiology, “Carol Davila” University of Medicine and Pharmacy, 050474 Bucharest, Romania; costin.caruntu@gmail.com (C.C.); cristian.scheau@umfcd.ro (C.S.); 8 Department of Dermatology, “Prof. N.C. Paulescu” National Institute of Diabetes, Nutrition and Metabolic Diseases, 011233 Bucharest, Romania; 9 Department of Oral and Maxillofacial Surgery, Faculty of Dental Medicine, “Titu Maiorescu” University, 031593 Bucharest, Romania

**Keywords:** cannabinoids, oral cancer, cannabis, palliative treatment, endocannabinoid system

## Abstract

Cannabinoids have incited scientific interest in different conditions, including malignancy, due to increased exposure to cannabis. Furthermore, cannabinoids are increasingly used to alleviate cancer-related symptoms. This review paper aims to clarify the recent findings on the relationship between cannabinoids and oral cancer, focusing on the molecular mechanisms that could link cannabinoids with oral cancer pathogenesis. In addition, we provide an overview of the current and future perspectives on the management of oral cancer patients using cannabinoid compounds. Epidemiological data on cannabis use and oral cancer development are conflicting. However, in vitro studies assessing the effects of cannabinoids on oral cancer cells have unveiled promising anti-cancer features, including apoptosis and inhibition of cell proliferation. Downregulation of various signaling pathways with anti-cancer effects has been identified in experimental models of oral cancer cells exposed to cannabinoids. Furthermore, in some countries, several synthetic or phytocannabinoids have been approved as medical adjuvants for the management of cancer patients undergoing chemoradiotherapy. Cannabinoids may improve overall well-being by relieving anxiety, depression, pain, and nausea. In conclusion, the link between cannabinoid compounds and oral cancer is complex, and further research is necessary to elucidate the potential risks or their protective impact on oral cancer.

## 1. Introduction

Oral cancer, the main type of head and neck cancer (HNC), represents a major global burden. Alongside the oral cavity, HNC encompasses malignancies developed from different structures of the upper aerodigestive system, including the lips, salivary glands, sinuses, oropharynx, and larynx [1]. The most common histological subtype of oral cancer is squamous cell carcinoma (OSCC), which represents more than 90% of these tumors [2]. According to the Global Cancer Observatory, the global incidence of OSCC continues to rise, reaching 377.713 new cases in 2020, and it is anticipated to increase by 30 percent in the next few decades [3,4]. Survival for oral cancer patients is dependent on many factors. By far, the most important factor is the disease staging at diagnosis and treatment. Thus, survival rates reach 86% when treatment is provided for incipient disease but can drop to 40% in the advanced stages of the disease [5]. Unfortunately, advanced disease at first presentation is a common finding in clinical practice [6]. An estimated half of the patients are diagnosed with locally or regionally spread disease [5], raising major challenges for the healthcare system. Significant costs are invested in the management of oral cancer patients, especially those with advanced disease, aiming not only to provide disease control but also to facilitate the independence and reintegration of the surviving patients in the community. Since the head and neck area is defined by highly complex anatomy with multiple functional and social roles, malignancies affecting this body region seem to be most distressing, with an important negative impact on functions such as eating, breathing, taste, speech, swallowing, and nevertheless esthetics [1]. Hence, oral cancer patients face significant difficulties in performing their daily activities and report significant impairment in their quality of life, with psychological and social consequences [7]. Furthermore, the treatment and overall management of oral cancer patients are a challenging, long-term, multi-disciplinary effort impacting medical and palliative care services and, overall, the global economy [8]. In the past decades, revolutionary surgical reconstruction techniques have been developed to allow the restoration of the integrity, function, and esthetic elements of the head and neck [9,10], including free microvascular tissue transfer [11], virtual surgical planning [12], and nonsurgical therapeutic strategies [13], but the results are still far from perfection. Moreover, progress in the technology allowing for early diagnosis was also recorded [14,15,16]. Thus, the scientific community continuously strives to find novel and highly efficient therapeutic strategies to help patients in need [17]. 

Being a risk factor-induced malignancy, many efforts are focused on strategies to minimize the general impact of oral cancer, including prevention strategies. The main behaviors exposing the population to the risk of developing oral cancer are tobacco and alcohol abuse, along with UV (ultraviolet) radiation for the lower lip subtype, poor oral hygiene, and human papillomavirus infection (HPV) [18,19]. Furthermore, tobacco has been shown to act synergically with alcohol abuse in increasing the risk of developing oral cancer [20]. Different carcinogen substances, such as nitrosamines found in cigarette smoke, acetaldehyde [21], urethane [22], or nitrosamines found in alcohol, especially after long-term consumption, have been associated with the development of pre-malignant or malignant lesions of the upper aero-digestive mucosa [23]. However, there are other types of substances that have been increasingly used by the population in the last decades, with unclear effects on the pathogenesis and progression of oral cancer [24]. Active compounds known as cannabinoids, found in different products, have raised attention in correlation with oral cancer pathogenesis [25]. The cannabinoids, as well as other phytochemicals such as phenolic compounds, have been increasingly studied throughout recent years and hold great promise in developing complementary, effective therapies [26,27,28].

The objective of this review article is to gather and analyze the most recent findings on the effects of cannabinoid compounds in the development and progression of oral cancer, focusing on the molecular mechanisms that could link cannabinoids with oral cancer pathogenesis. Furthermore, this paper aims to encompass the current knowledge and future perspectives on the therapeutic potential of cannabinoids and their role in the general management of oral cancer patients.

## 2. Endogenous and Exogenous Cannabinoids and Their Mechanisms of Action

Cannabinoids are a heterogenous group of active compounds produced naturally in our bodies—endocannabinoids, or those found in the plant called *Cannabis sativa* L.—phytocannabinoids [29], or synthetic cannabinoids, which are laboratory-produced compounds [30]. The endocannabinoid system (ECS) is a network of proteins, including receptors, their ligands, and degradative enzymes, that is widely distributed throughout the tissues and cells of mammals in virtually all animal species [31]. The ECS represents a crucial signaling pathway that plays a role in affective and cognitive functions [32], as well as in psychotic disorders [33], and might be the target for various therapeutic compounds. Moreover, the ECS is involved in the regulation of learning and memory processes [34], sleep [35], thermoregulation, pain control [36], inflammatory and immune responses [37], and it also plays a role in maintaining the body’s balance, triggering the desire to eat and feel hungry, and also promoting the storage of energy [38,39]. ECS comprises the following components: cannabinoid CB_1_ and CB_2_ receptors [40], endogenous agonists of these receptors—anandamide or N-arachidonoyl ethanolamine (AEA) and 2-arachidonoylglycerol (2-AG), and endocannabinoid metabolic enzymes—fatty acid amide hydrolase (FAAH) and monoacylglycerol lipase (MAG lipase) [41,42]. Recent evidence has suggested four new endocannabinoids [43]: N-arachidonoyl-dopamine (NADA) with main affinity for CB1 receptor [44], 2-arachidonyl glyceryl ether (noladin ether) with main affinity for CB1 and weak affinity for CB2 receptors [45], virodhamine (OAE) as a full agonist at CB2 and a partial agonist at CB1 and also acts as a CB1 antagonist in vivo [46], and Lysophosphatidylinositol (LPI), which activates GPR55 but not CB1 or CB2 [47]. 

CB1 and CB2 are members of the G-protein-coupled receptor (GPCR) family [48]. Their activation inhibits adenylyl cyclases and certain voltage-dependent calcium channels and activates several mitogen-activated protein (MAP) kinases [49], participating in signaling pathways involving cell proliferation, differentiation, survival, and apoptosis [50]. CB1 receptors are expressed mostly in the central nervous system as well as in some peripheral cells and are found with the highest density in the basal ganglia, substantia nigra, globus pallidus, cerebellum, and hippocampus, located on axon terminals and pre-terminal axon segments [50,51]. Also, the CB1 receptor, coupled through G_i/o_ proteins, modulates calcium conductance through N- and P/Q-type calcium channels, and previous studies have shown that it may also increase potassium conductance through an inwardly rectifying potassium channel [52]. CB1 receptors may also be expressed beyond the confines of the nervous system, for instance in the spleen, lung, thymus, heart, vasculature [43], and skin [53,54]. The CB2 receptor exhibits a notable presence in peripheral organs that possess immune-related functions, encompassing the spleen, tonsils, and thymus [55], in addition to being found in the lungs and testes [49]. The CB2 receptor is also present in immune cells, such as macrophages and leukocytes [56]. In terms of relative abundance, the CB2 receptor is found in decreasing order in tonsillar B cells > natural killer cells > monocytes and granulocytes > T4 lymphocytes > T8 lymphocytes [57]. Furthermore, CB2 receptors were initially identified in brain-resident immune cells, namely macrophages, and subsequently observed within neurons as well [58]. Additionally, there is a suggested third cannabinoid receptor known as G protein-coupled receptor 55 (GPR55), referred to as CB3 [59]. This receptor is thought to be one of the main receptors responsible for regulating vascular tone through the activation of cannabinoids [60]. GPR55 activation increases intracellular calcium [61]. It is generally accepted that, besides the CB_1_ and CB_2_ receptors, some endocannabinoids, phytocannabinoids, and several synthetic CB_1_/CB_2_-receptor agonists and antagonists can interact with various established non-CB_1_/non-CB_2_ GPCRs, ligand-gated ion channels, ion channels, and nuclear receptors [52]. 

Recent studies attempt to unveil the link between the pathogenesis of oral cancer and cannabinoids, investigating their potential as risk factors or their value as prognostic biomarkers, but also as therapeutic targets in the management of cancers [62,63]. 

Phytocannabinoids are found in *Cannabis sativa* L., an herbaceous annual plant cultivated in Central Asia almost 12,000 years ago and used in medicine and as a source of textile fibers [64]. The cannabis plant has come to attention due to its chemically active compounds: cannabinoids, terpenoids, flavonoids, and alkaloids [65], of which 1-delta-9-tetrahydrocannabinol (Δ9-THC) represents the primary psychoactive constituent [66]. More than 120 constituents produced in the glandular trichomes of the plant are terpenophenolic cannabinoids, or phytocannabinoids, with diverse chemical structures and pharmacological actions [67]. Besides Δ9-THC, the most studied phytocannabinoid, other components are Δ8-tetrahydrocannabinol (Δ8-THC), cannabinol (CBN), cannabidiol (CBD), cannabigerol (CBG) [41], and cannabichromene (CBC), Δ9-tetrahydrocannabivarin (THCV), cannabivarin (CBV), and cannabidivarin (CBDV). Despite their relatively lower abundance within the composition of the cannabis plant, several other phytocannabinoids, including cannabinodiol (CBND), cannabielsion (CBE), cannabicyclol (CBL), and cannabitriol (CBT), have garnered significant attention as subjects of investigation in recent decades [68]. 

One of the most intriguing and full-of-potential fields in the current study of cannabinoids is their capacity to regulate cell survival or death [69]. As a result, cannabinoids have the ability to induce proliferation, growth arrest, or apoptosis [69]. The specific effects of cannabinoids may vary depending on different experimental variables, such as the concentration of the drug, the timing of its administration, and the specific type of cell under examination [69].

## 3. The Effects of Cannabinoids on Oral Mucosa

Cannabis is the most frequently used illegal drug in Europe [70]. Approximately 48 million males and 31 million females have reported trying this substance [71]. The prevalence of marijuana use has exhibited a notable increase among young adults in recent years [72]. Cannabis, marijuana, and hashish are all derived from the cannabis plant, but there are differences between the three terms. Cannabis is the overarching term that refers to the plant itself and all of its products [73]. Marijuana specifically refers to the dried flower buds of the cannabis plant. Hashish, on the other hand, is a concentrated cannabis product made from the resinous parts of the plant. It is usually presented in the form of a paste and contains higher concentrations of psychoactive chemicals compared to marijuana, making it more potent and having stronger effects [29,30]. 

There are various ways to use cannabis, including smoking, vaping, and consuming it in food. Smoking cannabis can be done through a joint, blunt, or bong [74]. Additionally, cannabis can be consumed in food, such as edibles. Different modes of cannabis use are associated with demographic factors and other variables. Cannabis cigarettes are less tightly packed than tobacco cigarettes, and they are often smoked without filters or with smaller filters, resulting in higher smoke concentrations [75]. Cannabis smoke contains cancer-causing polyaromatic hydrocarbons similar to tobacco smoke but with a higher concentration, almost up to double the proportion due to the way cannabis cigarettes are smoked [76]. Phenols and polycyclic aromatic hydrocarbons are also present in the tar phase of marijuana smoke [77]. Marijuana tar has a 50% higher concentration of benzopyrene compared to unfiltered tobacco [77]. Compared to a filtered tobacco cigarette of the same weight, a marijuana cigarette leaves four times as much tar in the respiratory tract [78,79]. Prolonged inhalation may lead to the accumulation of tar particles in the oral cavity [78].

Cannabis use can cause oral cavity lesions such as cannabisstomatitis with leukoedema, hyperkeratosis, and leukoplakia [80]. Regular recreational use of hashish and marijuana is associated with a higher risk of severe periodontitis, including deeper probing depths and clinical attachment loss [81]. Gingival enlargement, similar to phenytoin gingival overgrowth, can also occur after marijuana use [82]. Furthermore, oral dryness was recorded among subjects who smoked marijuana, methaqualone, and tobacco [83]. Histological analyses show dysplastic changes in the buccal mucosa epithelium, including anucleated squamous cells, immature cell forms, increased nuclear pleomorphism, and abnormal mitotic activity [84,85]. The use of marijuana has been linked to various changes, such as lung conditions in humans, including chronic bronchitis symptoms, increased episodes of acute bronchitis, deoxyribonucleic acid (DNA) alteration, and abnormalities in the structure and function of alveolar macrophages [86,87]. Deep inhalation and breath-holding while smoking marijuana have been associated with pneumomediastinum, pneumothorax, and subcutaneous emphysema [88]. Also, a direct association was observed with the risk of upper and lower respiratory tract squamous cell cancers [89].

Recent studies showed that bacterial communities are different at various oral tumor sites from the adjacent normal tissue [90], and, more interestingly, the differences extend to the inner and outer surfaces of the tumor tissue [91]. Newmann et al. used 16s rDNA sequencing to investigate the effect of marijuana chronic exposure on the microbiome of the lateral border of the tongue and oral pharynx, common sites for oral cancer, and revealed changes in the bacterial communities with marijuana usage in these sites. However, the changes were consistent with malignancy only at the level of the pharynx [92]. Another recent study suggested a link between the development of squamous cell carcinoma of the tongue and the deregulation of the endocannabinoid system signaling pathway [93].

### 3.1. Cannabinoids and Oral Cancer

The relationship between smoking cannabis and the pathogenesis of oral cancer has been intensively studied in recent years (see Table 1). However, epidemiologic data connecting cannabis use and the development of head and neck malignancies is both inconsistent and contradictory [94].

#### 3.1.1. Reported Procarcinogenic Effects of Cannabinoids

Cannabis use was linked to a higher incidence of head and neck cancer, according to an early epidemiological study conducted by Zhang et al. [78]. This case-control study has observed a strong and consistent link between marijuana use and HNC among younger subjects and dose-response associations for both frequency and duration of marijuana use. Furthermore, the combination of marijuana smoking and cigarette smoking increased the risk of cancer, while alcohol consumption appeared as a negative cofounder [78]. In a review paper on oral cancer risk, Firth et al. described multiple case reports of young patients diagnosed with carcinoma of the upper aerodigestive tract and a confirmed history of marijuana use, raising the issue of its potential risk for OSCC development [95]. A very recent epidemiological panel regression study conducted in Europe identified 22 malignant disorders that showed a significant association with cannabis use, including cancers of the oropharynx, larynx, esophagus, stomach, colon, and rectum. Cannabis has been proven to be a stronger carcinogen compared to tobacco or alcohol. The primary factor associated with cannabis and its impact on cancer was found to be the concentration of the phytocannabinoid THC in the cannabis plant [96]. A different research study presents proof supporting a clear connection between smoking cannabis and a higher likelihood of developing laryngeal cancer [97]. This is explained by the fact that there is an overexpression of the epidermal growth factor receptor (EGFR) cascade in individuals with a history of cannabis smoking who have been diagnosed with laryngeal carcinoma [97]. A study aimed to establish the proportion of individuals who persisted in using tobacco or engaging in alcohol abuse even after being diagnosed for the first time with HNC or lung cancer. Additionally, the study aimed to identify the social, psychiatric, and addictive coexisting conditions that may contribute to maintaining unhealthy behavior. The study found that the patients who continued to smoke were usually using roll-up cigarettes, were drinking excessively in the past year, and had a high tendency to use cannabis prior to cancer diagnosis [98].

**Table 1:** Recorded effects of cannabinoids on oral cancer in recent in vitro and in vivo studies *.

Cannabinoid (Pure/Mix)	Method of Administration	Dose/Concentration	Experimental Model	Target Factors/Molecules	Effect	Reference
Procarcinogenic effects						
ACEA, Hu308, THC	Treatment of cell cultures	1 μM (ACEA), 1 μM (Hu308), 1 μM (THC)	UD-SCC-2, UPCI:SCC090, UM-SCC-104, and UM-SCC-47 (HNSCC cell lines)	p38 MAPK pathway	Increased cell proliferation	[99]
THC	Subcutaneous injection	3 mg/kg daily	HPV-positive HNSCC xenografts of nude mice	p38 MAPK pathway	Tumor progression	[99]
Cannabis	Smoking	N/A	Case-control cohort study (HNSCC)	N/A	Increased risk of primary SCC	[100]
Cannabis	Smoking	N/A	Ex vivo study (LSCC)	EGFR cascade	Increased expression of EGFR and downstream molecules	[97]
Marijuana	Smoking	More than 6 times ever	Case-control cohort study (HNSCC)	N/A	A nearly significant increase in the risk of cervical cancer	[101]
Marijuana	Smoking	20 or more days in the past month	Cross-sectional study (OSCC)	Oral microbiome	Changes in the oral microbiome consistent with malignancy	[92]
Marijuana	Smoking	N/A Dose-dependent	Case-control cohort study (HNSCC)	N/A	Increased dose-dependent risk of HNSCC incidence	[78]
Anti-carcinogenic effects						
Δ9, Δ8-THC	Treatment of cell cultures	IC50 = 10 μg/mL (Δ9-THC) and 13 μg/mL (Δ8-THC)	Ca9-22 oral cancer cells	Cyclin D1, p53, NOXA, PUMAα, DRAM, p21, H2AX	Decreased cell growth through apoptosis, autophagy, and oxidative stress	[102]
				E-cadherin	Suppression of migration/invasion	
CBD	Treatment of cell cultures	2 μM	Human oral squamous cell carcinoma, SAS, and human salivary gland cancer cells line ACCM	ID1, FOXM1, GDF15	Inhibition of tumor progression, stimulation of autophagy, suppression of migration/invasion	[103]
DHEA, EPEA, NALA	Treatment of cell cultures	10–30 µM	SNU-1041, SNU-1066 and SNU-1076	Akt	Apoptosis induced by increased 5-LO-mediated ROS production	[104]
Δ9, Δ8-THC	Treatment of cell cultures	Up to 200 μmol/L	Tu183 cell line	Mitochondria	Inhibition of mitochondrial oxygen consumption	[105]
Marijuana	Smoking	Moderate weekly use	Case-control cohort study (OSCC)	N/A	Decreased risk of HNSCC, regardless of HPV status	[106]
Marijuana	Smoking	≥50 hit-years	Case-control cohort study (OSCC)	N/A	Decreased risk of HPV-negative OSCC	[107]
Mixed or neutral effects on HNC carcinogenesis						
Marijuana	Smoking	Loose-leaf usage at least weekly	Human trial (HPV-related OPSCC)	N/A	No difference in survival, tumor recurrence, or adverse effects	[108]
Cannabis	Smoking	Any dose, frequency	Case-control cohort study (HNSCC)	N/A	No significant increase in the risk of developing HNSCC	[109]
Marijuana	Smoking	Any dose, frequency	Case-control cohort study (OSCC)	N/A	No association with an increased risk of OSCC	[110]

* ID1 = prometastatic gene inhibitor of DNA binding 1, FOXM1 = Forkhead box M1, GDF15 = growth differentiation factor 15, EGFR = epidermal growth factor receptor, LSCC = laryngeal squamous cell carcinoma, p38 MAPK pathway: the third major signaling cassette of the mitogen-activated protein kinase (MAPK) signaling pathway, HPV = human papillomavirus, HNSCC = head and neck squamous cell carcinoma, OSCC = oral squamous cell carcinoma, SCC = squamous cell carcinoma, THC = Tetrahydrocannabinol, CBD = cannabidiol, DHEA = docosahexaenoyl ethanolamide, EPEA = eicosapentaenoyl ethanolamide, NALA = N-arachidonoyl-L-alanine, Akt = protein kinase B, 5-LO = 5-lipoxygenase, ROS = reactive oxygen species, D1 = cyclin D1, p53 = cellular tumor antigen p53, NOXA = pro-apoptotic factor, PUMAα = mouse monoclonal IgG2b κ PUMAα antibody, DRAM = damage-regulated autophagy modulator, p21 = cyclin dependent kinase inhibitor, H2AX = histone H2AX tumor suppressor.

#### 3.1.2. Inconclusive Ties

A significant number of studies investigating the relationship between exposure to cannabinoids through cannabis use and the potential risk for HNC have failed to confirm a direct link between these two events, or even more, some studies have suggested a possible anti-cancer effect of cannabinoid compounds. The retrospective analysis of Sydney et al. on marijuana smoking and all cancer sites did not find any association between marijuana use and oral cancer occurrence. However, it did suggest a potential increase in the risk of prostate and cervical cancer among nonsmokers of tobacco cigarettes who use marijuana [101]. Rosenblatt et al. researched a larger sample of individuals who used marijuana and hashish and were exposed to various risk factors, such as smoking cigarettes, consuming alcohol, and having genetic polymorphism. Their findings indicated that there was no correlation between marijuana use and the development of oral cancer. All assessed parameters were generally comparable across groups, regardless of the marijuana consumption habits: regular versus infrequent use or ever use, and long-term versus short-term use. Furthermore, the study results showed no difference in oral tumor sites or staging in relation to the habits of marijuana use in the OSCC group of patients [110]. Similar work carried out by Aldington et al. on a smaller sample size, with 75 cases of HNC, did not find statistically significant links between cannabis use and the occurrence of HNC cancer among young adults. However, a significantly increased risk was reported in association with tobacco and alcohol consumption [109]. Another retrospective cohort study analyzed the link between cannabis use and the development of a second primary cancer in patients previously treated for HNC. The results suggest that phytocannabinoids found in cannabis do not have carcinogenic effects and do not induce changes in the field of cancerization [101]. 

The meta-analysis undertaken by Ghasemiesfe et al. examined the correlation between marijuana usage and the occurrence of cancer in adults who have been exposed to at least one joint year. The study found no evidence of a connection between marijuana use and the likelihood of developing OSCC. The interpretation of these findings should be considered with care due to the insufficient quantification of marijuana use and inadequate adjustment for confounding factors [111]. The International Head and Neck Cancer Epidemiology (INHANCE) consortium conducted one of the most extensive studies on the link between smoking marijuana and the risk of HNC. Specifically, they focused on individuals with a negative history of smoking tobacco or alcohol. The study results did not confirm the increased risks for HNC based on marijuana use, regardless of the occasional or frequent consumption, duration of use, cumulative use, or specific subsites of cancer. This was true for both individuals who never used tobacco and those who never used tobacco or alcohol. While the results suggested an association between marijuana smoking and oropharyngeal cancer, it was not confirmed through a dose-response relationship [112]. Vaping phytocannabinoid compounds, a more recent alternative to smoking marijuana, has gained popularity because, when heated to 180–200 °C and then vaporized, cannabis releases a significant quantity of THC with just trace levels of other compounds [113]. Vaping marijuana is thought to have fewer long-term toxic effects compared to smoking, but there is insufficient evidence to support this statement [114]. When cannabis is vaporized, fewer smoke-related chemicals and carcinogens, including carbon monoxide, tar, ammonia, and hydrogen cyanide, are inhaled [115]. The impact of vaping through electronic cigarettes on oral health remains unclear and is a subject of ongoing debate among scientists and clinicians. Limited research has been conducted on the potential toxic effects of electronic nicotine delivery systems (ENDS) or e-cigarette aerosols on oral cells. Additionally, the available clinical studies in this area are still in the preliminary stages and have small sample sizes. Although e-cigarette vapor contains lower levels of carcinogenic substances such as formaldehyde, acetaldehyde, and acrolein compared to cigarette smoke [116], it still includes harmful constituents. Additionally, e-cigarette vapor may also contain considerable amounts of potential carcinogenic toxic metals like aluminum, cadmium, chromium, and copper [117]. This suggests that vaping is not quite a reliable method of cannabinoid delivery, also sustained by the fact that the outbreak of electronic-cigarette, or vaping, product use–associated lung injury (EVALI) was associated in most cases with THC product fluids containing vitamin E acetate [118,119].

#### 3.1.3. Anti-Carcinogenic Impact

A recent meta-analysis examined the association between exposure to cannabinoids through cannabis use and cancer risk in the United States population. The analysis included 34 studies with 55 data points and reported a trend toward reduced risk for cancer in cannabis users. However, the results have to consider the high heterogeneity of the study. In HNC, a negative association with cannabis use was reported [120]. The study highlighted the potential anti-tumor effects of cannabinoids. Phytocannabinoids have been found to reduce obesity, inhibit chronic inflammation, lower fasting insulin levels and insulin sensitivity, and thus possess direct anti-tumor effects [120]. The anti-tumor activity of cannabinoid compounds is further discussed and detailed in Section 4.

However, caution must be exercised when interpreting these findings regarding the potential risk or protective impact of phytocannabinoids on HNC due to the presence of confounding factors such as tobacco and alcohol, usually encountered in recreational use. More studies are necessary to determine the direction of phytocannabinoids in cases of only cannabinoid users.

### 3.2. The Relationship between Cannabinoids and HPV-Positive HNC

Viruses play an important role in cancer pathogenesis. The Epstein–Barr virus DNA is found in most of the nasopharyngeal cancer specimens, while human papillomavirus (HPV) contributes to developing specific types of HNC [109,121]. The correlation between HPV infection and oropharyngeal squamous cell carcinoma cancer, particularly tumors emerging from the lingual and palatine tonsils, was investigated by Gillison et al. Both cancer sites are etiologically related to infection with high-risk strains of HPV, particularly HPV16, and reveal a higher basaloid prevalence and fewer p53 mutations in tumor specimens. Furthermore, the correlation with alcohol abuse and cigarette use is of reduced significance, and patients have an apparent higher survival rate [122]. The authors extended their research on HPV infection and investigated other potential risk factors, including cannabis use. In a cross-sectional study on the prevalence of oral HPV infection in the United States, several demographic and behavioral habits have revealed associations with HPV infection. Thus, an increased prevalence of oral HPV-16 infection, as well as overall oral HPV infection, was reported in male subjects, current and former marijuana users, tobacco smokers, and heavy alcohol users [123]. The oncoproteins E6 and E7 of HPV have an impact on various cellular functions and molecular signaling pathways. This includes the inactivation and breakdown of the tumor suppressor protein p53 and the retinoblastoma (RB) protein [124], as well as the deactivation of cyclin D1, leading to procarcinogenic effects [125]. The phytocannabinoids have a similar effect on molecular signaling pathways, downregulating the expression levels of cyclin D1 and protein p53 [102].

Chao Liu and colleagues conducted a study on the role of cannabis in the development of HPV-positive head and neck squamous cell carcinoma (HNSCC) and its interaction with cannabinoids. They discovered that the levels of CB1 mRNA and CB2 mRNA were significantly higher in HPV-positive HNSCC compared to HPV-negative HNSCC. When they decreased the expression of CB1 and CB2 in HPV-positive HNSCC cell lines, they observed a significant reduction in cancer cell proliferation. On the other hand, when they treated HPV-positive HNSCC cell lines with selective CB1 agonist ACAE, selective CB2 agonist Hu 308, and non-selective cannabinoid receptor agonist THC at doses similar to those experienced by marijuana users for recreational purposes, they observed an increase in cell proliferation compared to normal cell lines. Additionally, they used rimonabant, a selective CB1 antagonist, and SR144528, a CB2 antagonist, to block cannabinoid receptors in HPV-positive HNSCC and found that these receptor antagonists not only inhibited cell proliferation but also triggered apoptosis in HPV-positive HNSCC cells [99].

Particular risk-factor profiles for HPV-positive versus HPV-negative oral and oropharyngeal squamous cell carcinoma (OPSCC) were examined in a recent case-control study concerning demographic and behavioral elements, including marijuana use. The results concluded that marijuana use does not correlate with HPV-positive cancer occurrence and reported a negative correlation with HPV-negative cancer [107]. Another population-based study found a reduced risk for HNSCC in subjects with long-term exposure to phytocannabinoids who reported between 10 and 20 years of marijuana smoking. This finding was not dependent on HPV-16 antibody status. The amount of marijuana used did not impact the risk of cancer. Additionally, it was found that marijuana interfered with the effects of alcohol and tobacco abuse, lowering the risk for HNSCC in moderate and light drinkers while elevating it in heavy smokers and drinkers [106]. 

Studies have shown that a younger age and modified sexual behavior [126] impact the overall risk of HPV-positive HNC [127,128]. Furthermore, Sun et al. reported a positive correlation between marijuana use and sexual frequency in all demographic groups included in their study [129]. A cross-sectional study of 735 Hispanic adults analyzed marijuana usage to assess the association between marijuana use and periodontal disease and oral HPV infection as potential risk factors for OPSCC. Their results showed that there is a link between marijuana use and severe periodontal disease, but they could not correlate marijuana use with oral HPV infection [130]. In a recent case-matched cohort study, the survival outcomes of HPV-positive OPSCC were examined, taking into account different criteria, including marijuana use. The study included 47 current marijuana users with P16-positive OPSCC treated with curative intent and 47 control patients with no exposure to marijuana. Their results found no significant differences between marijuana users and non-users in terms of disease-specific, disease-free, and metastasis-free survival in patients with HPV-positive OPSCC [108].

The aforementioned studies failed to identify a connection between marijuana use and HPV-positive HNC, both of which were strongly confounded by sexual behavior. Preliminary data suggests that long-term marijuana use could provide some protective effects against the risk of HNC. This finding aligns with the potential anti-carcinogenic properties of cannabinoid compounds.

## 4. In Vitro Experimental Models on the Anti-Tumor Activity of Cannabinoid Compounds

**Figure 1:**
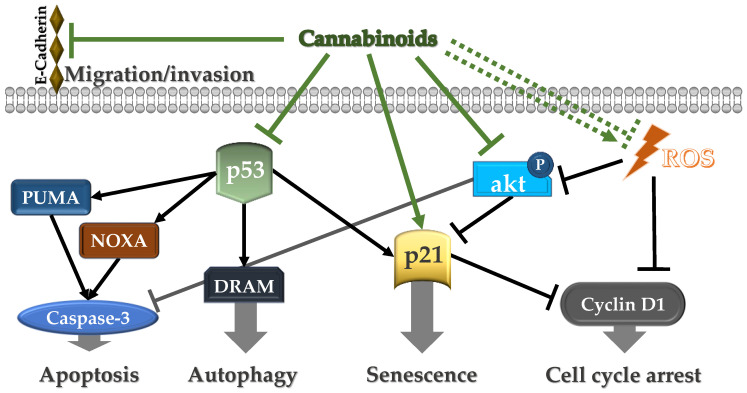
Molecular mechanisms of cannabinoids on cancer cells. ROS = reactive oxygen species, D1 = cyclin D1, p53 = cellular tumor antigen p53, NOXA = pro-apoptotic factor, PUMAα = mouse monoclonal IgG2b κ PUMAα antibody, DRAM = damage-regulated autophagy modulator, p21 = cyclin dependent kinase inhibitor, Akt = protein kinase B.

Researchers have investigated the effects of endocannabinoids on several cancer cell types in experimental environments (see Figure 1). Even though some pathways have been unveiled, the exact mechanisms underlying the anti-cancer effects of cannabinoids are still unknown. Anandamide (AEA), the endogenous agonist of the cannabinoid receptors, has been shown to cause cellular death in an apoptosis-resistant colorectal cancer cell line overexpressing cyclooxygenase-2 (COX-2) [131]. AEA induced apoptosis of uterine cervical carcinoma cells by targeting the vanilloid receptor-1 [132] and induced cell death in the PC-12 cell line [133] by activating the p38 MAPK, c-Jun N-terminal kinase (JNK), and p44/42 MAPK pathways via a cannabinoid/vanilloid receptor-independent pathway [134]. Various other components of the immune system may be activated during carcinogenesis, and an interplay with inflammation was also shown to exist [135,136].

Concern may exist regarding the use of endocannabinoids as a cancer therapy because it has been revealed that they mediate the psychotropic side effects of cannabis through the traditional cannabinoid receptors CB1 and CB2 [137]. However, it has also been shown that the ability of certain endocannabinoids, including anandamide, the endogenous structural analog of anandamide, N-arachidonoyl glycine (NAGly), as well as the related polyunsaturated fatty acids arachidonic acid and eicosapentaenoic acid, to induce apoptosis is mediated by pathways not necessarily involving CB receptors [138].

In a study on HNSCC cell lines, Park et al. investigated the impact of docosahexaenoyl ethanolamide (DHEA) and N-arachidonoyl-L-alanine (NALA), two polyunsaturated fatty acid (PUFA)-based ethanolamides with a chemical structure similar to AEA [139] (both endocannabinoids) [104]. The objective was to explore their anti-cancer properties and mechanisms of action. Both DHEA and NALA effectively inhibited cell proliferation through 5-LO-mediated ROS production in a receptor-independent manner, even though HNSCC cells may express specific receptors, such as CB1 and/or TRPV1 [132]. 5-Lipoxygenase (5-LO) is an enzyme involved in arachidonic acid (AA) metabolism [140], which has a chemical structure similar to several endocannabinoids [141]. Furthermore, DHEA and NALA decreased the phosphorylated form of Akt by increasing intracellular ROS production [104]. Evidence from previous research suggests that the production of ROS disrupts the regulation of the redox system and promotes the process of carcinogenesis [142,143]. The PI3K/Akt/mTOR (Phosphoinositide 3-kinase/Protein kinase B/mammalian target of rapamycin) pathway plays a crucial role in regulating cell metabolism, growth, and survival in normal physiological conditions [144] and in many pathological conditions, including digestive cancers [145]. However, in HNSCC, various cancer-related characteristics such as immune suppression, inflammation, angiogenesis, survival, invasion, and metastasis are associated with abnormalities of this pathway [146,147]. Currently, pharmaceutical companies and academic institutions are actively involved in developing and studying inhibitors targeting PI3K, Akt, and mTOR for the treatment of HNSCC in both preclinical and clinical settings [148].

A recent study by Semlali et al., aimed to investigate the impact of Δ9-THC and Δ8-THC on the behavior of a human gingival squamous carcinoma cell line known as Ca9-22 [102]. Their findings suggest that these compounds could be used as potential therapy agents for oral cancer. It was observed that 10 or 20 μM of either Δ9-THC or Δ8-THC exhibited an inhibitory effect on the proliferation of cancer cells. The effects of Δ9-THC and Δ8-THC on the oral cell line included a decrease in cell viability and proliferation, activation of the apoptotic pathway through increased caspase 3 activity starting from exposure to 10 μM, and induction of S and G2/M cell cycle arrest in Ca9-22 cells. Additionally, other mechanisms were identified that support the idea of utilizing cannabinoids as a treatment for oral cancer. These mechanisms include cell autophagy, suppression of migration and invasion, inhibition of oxidative stress, and improvement of antioxidant activity. Furthermore, Δ9-THC demonstrated greater effectiveness compared to Δ8-THC. The study also found that the expression levels of cyclin D1, p53, NOXA, PUMA, and DRAM were downregulated by Δ9-THC and Δ 8-THC, while p21 and H2AX expression were upregulated. These results confirm that cannabinoids effectively inhibit various signaling pathways associated with oral cancer cells, including the MAPK/NF-κB and Wnt/β-catenin pathways [102].

Further research has shown promising results regarding the anti-tumor activity of Δ9-THC and Δ8-THC on cancer cell lines that are highly resistant to cytotoxic drugs. In a study conducted on Tu183 cells, derived from a squamous cell carcinoma of the tonsils [149], the impact of different doses of Δ9-THC and Δ8-THC on cellular respiration was assessed. The findings revealed a rapid and potent inhibition of cellular mitochondrial O_2_ consumption after exposure to these compounds, surpassing the effects of commonly used anti-cancer medications. Interestingly, the ineffectiveness of AEA suggests that the effects of Δ9-THC and Δ8-THC are not mediated by the cannabinoid receptor pathways [105]. 

By making efficient use of the characteristics of endocannabinoids and phytocannabinoids, there is a possibility to develop innovative derivatives that specifically target HNC without inducing the activation of cannabinoid receptors and avoiding the occurrence of psychoactive side effects.

## 5. Cannabinoids in the Management of Oral Cancer-Related Symptoms

The first state to allow the medical use of cannabis since it was federally prohibited in 1973 was California State on 5 November 1996 [150]. To date, more than 40 countries have legalized cannabis, fully or partially, for medical and/or recreational use [151]. The acceptance of medical cannabis is even more widespread [152], encompassing 38 states in the United States, Canada, and a significant portion of Europe (Croatia, Denmark, Greece, Germany, Italy) [151], and Australia [153,154]. Notably, in 2021, Malta became the first member state of the European Union to permit the possession and cultivation of recreational cannabis within specific limits [154].

Marijuana’s special capacity to promote relaxation, relieve anxiety, and reduce discomfort is well documented [155]. In recent times, physicians have taken notice of the potential medical uses of marijuana (see Table 2), especially after the discovery of the ECS in humans, which has sparked increased interest in exploring its potential applications [89]. The term ‘medical marijuana’ is somewhat ambiguous, as it can encompass different forms in which cannabinoids exist. These forms include phytocannabinoids and synthetic cannabinoids, laboratory-produced in relation to THC and CBD chemical structures, serving as the foundation for the pharmaceutical industry’s cannabinoid-related products [156]. Medicinal cannabis is available in a variety of forms, each containing different concentrations of the two primary therapeutic cannabinoids: tetrahydrocannabinol (THC) and cannabidiol (CBD) [157]. It can be consumed as tablets, sprays, creams, edible products (brownies, cookies, candies, gummies, chocolate bars, cakes, tinctures, and beverages), and oils. Additionally, some cancer patients use cannabis leaves or buds to create other forms of administration like oils or oral solutions, edibles, suppositories, or topicals. They may obtain these products legally, through licensed suppliers, or illegally [158].

THC has the ability to attach to both CB1 and CB2 cannabinoid receptors, although it has been proven to have a lower inherent attraction to CB2 compared to CB1 [159]. It has been shown to increase appetite and promote weight gain. Additionally, THC has a strong ability to reduce acute and chronic pain, as well as help alleviate symptoms of multiple sclerosis, such as muscle spasticity [160].

**Table 2:** Applications of cannabinoids in the management of cancer-related symptoms in head and neck cancer patients.

Cannabinoid (Pure/Mix)	Method of Administration	Dose/Concentration	Study Category (Cancer Type)	Population Size	Beneficial Effects	Adverse Effects	Reference
Marijuana	Smoking	Loose-leaf usage at least weekly	Cohort study (OPSCC, OC, HP, L)	74 patients, 74 controls	Decrease in anxiety/depression, pain/discomfort, and tiredness. Increase in appetite and well-being	N/A	[161]
Medical Marijuana	N/A	N/A	Cohort study (HNC)	63 patients	Decrease in headache, pain, nausea, loss of appetite, and reduced anxiety	N/A	[162]
Marijuana	Smoking	Loose-leaf usage at least weekly	Case-control (P16-positive OPSCC)	47 subjects and 47 controls	There is no survival difference.	No effects	[108]
Nabilone	Ingested	0.5 mg	Randomized Double-Blind (HNC)	56 patients	No beneficial effects	No effects	[163]
Medical Marijuana	Inhalation, ingestion, and oil.	Various dosages; the majority used daily or more than once daily	cross-sectional survey (HNC RT)	15 patients	Decrease in anxiety/depression, pain, and maintaining weight. Improving appetite, swallowing, xerostomia, and relieving muscle spasms	N/A	[164]
Marijuana	N/A	N/A	retrospective cohort study (OPSCC)	74 patients	No beneficial effects	lower overall survival rates and greater weight loss during radiotherapy.	[165]

OC = oral cavity, HP = hypopharynx, L = larynx, N/A = not applicable, OPSCC = oropharyngeal squamous cell carcinoma, RT = radiotherapy; HNC = head and neck cancer; P16-positive = tumor suppressor protein that inhibits cyclin-dependent kinase 4A.

Currently, two synthetic Δ9-THC compounds have undergone clinical trials and received approval for alleviating nausea and vomiting in individuals undergoing cancer chemotherapy. These compounds are known as nabilone and dronabinol [166]. In Canada, nabiximols, which contain approximately equal amounts of THC and CBD, have been granted approval for the treatment of pain associated with cancer [167]. Medical cannabis can be utilized as a supplementary approach to conventional pain management for both short-term and long-term pain [168]. Medical cannabis can be introduced to patients who are undergoing active treatment or who are unable to effectively utilize standard pain medications [169]. However, in oral cancer patients, a recent systematic analysis of the utilization of cannabis and cannabinoids as supplementary treatments yielded mixed outcomes [29]. Another cohort study conducted on newly diagnosed HNSCC patients who were current marijuana users and were undergoing curative treatment found that recreational use of Cannabis sativa had the potential to relieve anxiety, depression, pain, and nausea while also improving their overall well-being [161]. A research study was conducted to explore the perspectives of marijuana use in the general management of HNC patients. Individuals who had previously used or were currently using marijuana reported experiencing relief from symptoms such as headaches, pain, nausea, and loss of appetite. A significant majority, 83% of all patients, viewed marijuana as a potential treatment for pain associated with cancer, while 67% considered it a possible remedy for cancer-related anxiety [162]. On the other hand, Ghanem et al. focused on the effects of marijuana use/abuse on survival and local disease control in patients with OPSCC and found that the use of cannabis was associated with lower overall survival rates and greater weight loss during radiotherapy in these patients. However, no significant differences were reported regarding disease-free status, loco-regional control, distant metastasis, or pain score [165].

The current body of literature examining the utilization of cannabinoids in patients receiving radiation therapy (RT) is significantly limited. The use of cannabinoids has the potential to alleviate anxiety in patients before commencing RT and diminish the occurrence of nausea and vomiting, in line with current standard treatment practices. It may also provide relief from symptoms after three years following RT for HNC, although these effects do not occur during or immediately after the treatment [170]. In a separate investigation, individuals who had previously been diagnosed with HNC and underwent RT expressed various advantages to the use of medical marijuana. These benefits included managing anxiety, maintaining a healthy weight, alleviating depression and pain, improving appetite, addressing difficulties with swallowing, reducing dry mouth, relieving muscle spasms, and managing viscous saliva production [164]. In a randomized controlled trial conducted to evaluate the effects of the synthetic cannabinoid, nabilone, at a dose of 0.5 mg administered orally in patients with HNSCC undergoing RT, the results showed that nabilone was well-tolerated and did not cause any noticeable negative effects such as increased drowsiness, anxiety, or dry mouth. However, no significant improvements were found in appetite, pain, sleep control, or overall quality of life [163]. A recent systematic review investigating the advantages of cannabis-based medications for alleviating pain and other symptoms in adult cancer patients compared to placebo or other analgesics reported limited evidence supporting the effectiveness of nabilone. Over eight weeks, nabilone did not outperform the placebo in reducing pain associated with chemotherapy or radiochemotherapy in individuals with HNC. Additionally, it revealed that oromucosal nabiximols and THC are not effective in relieving moderate-to-severe opioid-refractory cancer pain [171].

CBD, the primary non-psychotropic element, exhibits a low affinity for CB1 receptors. It stimulates receptors that regulate fear-related and psychological stress responses. CBD possesses anxiolytic, antidepressant, antipsychotic, anticonvulsant, antinausea, antioxidant, anti-inflammatory, anti-arthritic, and antineoplastic properties [160]. Literature data on pure CBD as a treatment for oral cancer and cancer-related symptoms in head and neck cancer patients is very scarce. Research has shown that CBD may offer protection against the onset of oral mucositis, a frequent side effect of radiation therapy for the head and neck region, because of its antioxidant, anti-inflammatory, and analgesic qualities [172]. CBD can be used according to a clinical trial to prevent taste alteration, a well-known side effect of cancer therapy [173].

Thus, it is important for oncology care providers to familiarize themselves with the different routes of administration, dosages, and potential risks associated with medical cannabis. This knowledge will enable them to make informed recommendations. Further studies on the link between marijuana use and malignancies of the mouth, lungs, and other organs through long-term studies of marijuana-only users would be highly beneficial.

## 6. Discussion and Conclusions

The relationship between cannabinoids and the pathogenesis of oral cancer is complex and marked by contradictory findings. Early epidemiological studies suggest a higher incidence of head and neck cancers linked to marijuana use, particularly among younger individuals, with dose-response associations [78,95,100]. In the initial studies, tobacco smoking and alcohol were identified as cofactors. Recent research has identified significant associations between cannabis use and various malignant disorders, including oropharyngeal cancer [96]. Cannabis is a stronger carcinogen than tobacco or alcohol, with the concentration of the phytocannabinoid THC playing a key role [96]. Other studies do not support a direct association or even suggest potential anti-cancer effects of cannabinoids in oral cancer [101,110,111].

The underlying mechanisms have been investigated through in vitro studies revealing that endocannabinoids could induce cellular death through the activation of different pathways, including p38 MAPK, JNK, and p44/42 MAPK [134], or can inhibit cell proliferation in a receptor-independent manner by increasing intracellular ROS production and suppressing the PI3K/Akt/mTOR pathway [104]. However, concerns have been raised about the psychotropic side effects mediated through the CB1 and CB2 receptors of endocannabinoids. In experimental models, Δ9-THC and Δ8-THC have revealed encouraging effects on oral cancer cell lines by inhibiting cell proliferation, activating apoptosis, inducing cell cycle arrest, suppressing migration and invasion, and affecting various signaling pathways [102]. These phytocannabinoids also demonstrated effectiveness against cytotoxic drug-resistant cancer cell lines by inhibiting cellular mitochondrial O_2_ consumption [105]. 

In therapeutic applications, medical marijuana containing cannabinoids is legalized in numerous countries for its potential to alleviate cancer-related symptoms [155,166]. Medical cannabis can be used as a supplementary approach to conventional pain management, especially for patients undergoing active treatment [168,169]. However, the utilization of cannabis as a supplement for oral cancer patients yielded mixed outcomes. While some studies showed potential benefits in relieving anxiety, depression, pain, and nausea [161,162,164], others found associations with lower overall survival rates and greater weight loss [165]. However, studies evaluating the effects of synthetic cannabinoids did not show significant improvement in pain management [171].

In essence, while some studies suggest associations between cannabis use and head and neck cancers, the overall landscape remains inconclusive and calls for further investigations to elucidate the intricate interplay between cannabis, its constituents, and oral cancer risk. The diverse findings highlight the need for nuanced considerations, recognizing the multifaceted nature of individual behaviors, coexisting factors, and evolving methods of cannabis consumption.

## Data Availability

Not applicable.
